# Stress-Induced Cross-Protection and Combined Stress Responses in Extremotolerant Black Yeasts

**DOI:** 10.3390/jof12010043

**Published:** 2026-01-06

**Authors:** Klavdija Fortuna, Maja Kajin, Cene Gostinčar

**Affiliations:** Department of Biology, Biotechnical Faculty, University of Ljubljana, Jamnikarjeva 101, SI-1000 Ljubljana, Slovenia; klavdija.fortuna@bf.uni-lj.si (K.F.); maja.kajin@bf.uni-lj.si (M.K.)

**Keywords:** *Aureobasidium*, *Hortaea werneckii*, ploidy, salt stress, temperature stress, combined stress effects, stress preconditioning, freezing survival, desiccation survival

## Abstract

Extremotolerant fungi inhabit environments with multiple overlapping stressors, yet most studies examine stresses individually. We tested whether preconditioning with salt, cold, or both improves survival after desiccation and freezing, and whether combined salinity and temperature effects on growth are additive or synergistic. We studied *Aureobasidium pullulans*, *Aureobasidium subglaciale*, *Aureobasidium melanogenum*, and *Hortaea werneckii* (haploid and diploid). All preconditioning treatments significantly increased long-term desiccation survival in *A. pullulans*, reflecting its generalist capacity to activate cross-protective responses. *H. werneckii* displayed smaller improvements, consistent with a specialist strategy. Freezing survival without cryoprotectants remained ~100% in both species, indicating high intrinsic tolerance. Growth analyses revealed synergistic effects of salinity and temperature in *Aureobasidium* spp. Species differed in salinity sensitivity (*A. melanogenum* > *A. pullulans* > *A. subglaciale*) and thermal preferences. *A. melanogenum* and *A. pullulans* grew faster at higher temperatures, while *A. subglaciale* showed the opposite trend. In *H. werneckii*, salinity governed growth. Haploids slowed as salinity increased, while the diploid remained unaffected. This is the first confirmation of the long-standing suggestion that hybrid diploid genomes of many *H. werneckii* are an adaptation to osmotic stress. These findings illustrate two pathways to extremotolerance: inducible flexibility in *Aureobasidium* versus constitutive halotolerance in *H. werneckii*.

## 1. Introduction

Environments such as glaciers, hyperarid deserts, and hypersaline lakes are considered “extreme” due to their physical and chemical hostility to life [1]. Nevertheless, microbial life is consistently detected in these habitats. Once thought to be dominated by prokaryotes, extreme environments are now known to host a diversity of extremotolerant fungi with unique adaptations in physiology, morphology, reproduction, and other aspects [1,2,3,4,5,6,7,8,9,10,11]. Among extremotolerant fungi, black yeasts are of particular interest due to their ability to withstand diverse environmental stressors [1,5,8,9,12,13,14,15,16,17,18,19]. Studies of extremotolerance have focused on a handful of black yeast species, including *Aureobasidium* spp. and *Hortaea werneckii* [4,5,8,13,20].

*Aureobasidium pullulans*, the type species of the genus (Ascomycota, Dothideomycetes, Dothideales, Dothioraceae) [17], is a polyextremotolerant generalist with a high degree of phenotypic plasticity [13]. It occurs in diverse habitats [12], including glacial ice [21,22], hypersaline salterns [23], plant surfaces [24], food [25], and even indoor anthropogenic environments [26,27]. It tolerates up to 17% NaCl (*w*/*v*), and survives in acidic, basic [28,29], cold [30,31], and oligotrophic environments [21]. Industrially, it is exploited for its production of pullulan, extracellular enzymes, and as a biocontrol agent [22,32]. Other species in the genus include *A. subglaciale* and *A. melanogenum* [12,22]. *A. subglaciale* is psychrophilic, primarily isolated from cold habitats [12], and tolerates high salinity, UV and gamma radiation, and heavy metals [12,21,22,33,34,35]. *A. melanogenum* is common in aquatic and anthropogenic settings such as tap water and dishwashers, displaying strong halotolerance and melanisation. Unlike other *Aureobasidium* spp., it grows at 37 °C, suggesting an increased potential for opportunistic pathogenicity [12,22].

In contrast to the generalist *A. pullulans*, *Hortaea werneckii* (Ascomycota, Dothideomycetes, Capnodiales, Teratosphaeriaceae) [9,36,37] is a halotolerant specialist. Although sodium chloride is not essential for its growth, it can thrive in almost saturated NaCl solutions, with an optimum between 5 and 10% (*w*/*v*) (or 0.8 M and 1.7 M) NaCl [9]. This ability places *H. werneckii* among the fungi with the widest known halotolerance ranges [1,14,15,37,38]. It is distributed worldwide [38], but is primarily associated with hypersaline environments [39], including solar salterns [40] and other adjacent habitats, such as sea sponges, marine and salted freshwater fish, corals, microbial mats in salterns, beach soil, salt marsh plants, and salted food [2,14,39,41,42,43]. Genomic studies revealed that the wild population of *H. werneckii* contains both haploid isolates and stable, highly heterozygous diploid isolates that originate from several independent hybridization events between phylogenetically distant haploid lineages. Despite the presence of a homothallic mating type locus, population genomic and linkage analyses indicate that the species reproduces clonally, and the mechanism of the observed hybridization remains unknown. Hybrid diploids represent more than half of natural isolates. Although this unusual reproductive strategy has been suggested to play a role in the extremotolerance of *H. werneckii*, evidence supporting such a role has so far not been found [20].

Organisms inhabiting extreme environments such as salterns, glaciers, and hyperarid deserts often encounter similar challenges. *H. werneckii* and *Aureobasidium* spp. are among the few fungi capable of tolerating such conditions, primarily due to their ability to survive in environments with low water activity (a_w_). Water activity denotes the fraction of biologically available water, which is reduced either by the presence of solutes, such as salts, or by freezing and desiccation. Low a_w_ causes water to leave the cell, reducing turgor pressure and dehydrating the cytosol. Consequently, intracellular solutes become more concentrated, and the influx of inorganic ions can further disrupt membranes and damage intracellular structures [1,5,9,21,37]. *H. werneckii* and *Aureobasidium* spp. counter these effects by synthesizing and accumulating compatible solutes (e.g., glycerol, arabitol, mannitol), and in the case of *H. werneckii* also mycosporines and mycosporine-like amino acids [15]. These maintain osmotic balance, prevent loss of turgor and limit cytosolic dehydration [1,9,18,21,44,45]. Glycerol is the primary solute in both species; its retention is supported by transporters [18,44,46], adjustments of membrane composition [21,47], and melanin-rich cell walls [15]. Glycerol can be recycled as a carbon source when the stress subsides [46]. Additionally, compatible solutes (including glycerol) also function as cryoprotectants that mitigate mechanical damage caused by intracellular ice crystals, a unique challenge of freezing that is not present in other low a_w_ conditions [13,21,43]. Some compatible solutes might also act as non-enzymatic scavengers of reactive oxygen species (ROS), which are produced during stress [21,48,49,50]. Small metabolites, low-molecular-weight manganese complexes and small peptides have been shown to act as ROS scavengers in a number of organisms. Their action complements the enzymatic antioxidants, which primarily include superoxide dismutases, catalases, and peroxiredoxins [21].

The intracellular environment of halotolerant black yeast differs significantly from the extracellular environment, meaning that the intracellular machinery is not adapted to extreme salt concentrations, as it is never exposed to them [9]. The two exceptions are the plasma membrane and the cell wall, both of which respond to hypersaline conditions. Membrane composition is adjusted via sterol and fatty acid remodelling [47] while the cell wall, especially in *H. werneckii*, is highly melanized and has low chitin content [15,21,51,52,53]. Ion homeostasis relies primarily on Na^+^ and K^+^ transporters [9,54]. These adaptations are energy-intensive, triggering upregulation of central metabolic pathways and mitochondrial biogenesis, which may lead to increased oxidative stress [48,49,55,56]. *A. pullulans* is capable of entering anhydrobiosis, a state of suspended metabolism under extreme desiccation [21]. While halotolerance is a complex, polygenic trait involving genes from multiple pathways and regulatory networks, rare examples of pronounced single-gene contributions have been documented, in which modification of a single locus can disproportionately influence salt tolerance [57].

Morphological adaptations accompany these molecular strategies. Filamentous growth supports nutrient exchange but increases water loss due to a high surface-to-volume ratio [58,59], whereas yeast cells function as individual units with greater protection from the cell wall. Beyond this dichotomous classification, black yeasts exhibit morphological plasticity, expressed through a wide range of unusual morphologies, including budding, fission, lateral conidia, endoconidiation, and meristematic growth, often co-occurring within a single culture. This enables them to harness the advantages of each growth form depending on environmental conditions [17,22,60]. Meristematic clumps, composed of slow-growing, densely packed cells, minimize surface exposure and protect internal cells from environmental stress [61,62]. Biofilms form to enable surface attachment and to prevent water loss [1]. In *H. werneckii*, meristematic hyphae may also act as dormancy structures in saline environments [63,64]. The polymorphism of the generalist *A. pullulans* under stable conditions likely reflects a bet-hedging strategy for environmental variability [65], unlike the more limited plasticity of *H. werneckii* [36].

Preconditioning, or stress priming, is a widely conserved phenomenon reported across all kingdoms of life, from bacteria and fungi to plants and animals. Mild exposure to one stress can increase resilience to subsequent, often unrelated challenges, providing cross-protection through inducible stress-response pathways [66,67,68,69,70,71]. The existence and extent of stress priming in extremotolerant black yeasts such as *A. pullulans* and *H. werneckii* remain unknown. Similarly, the knowledge on combined stress responses in these organisms is scarce, as most available literature has focused on the effects of a single source of stress studied in isolation [49,51,64].

In this study, we investigated whether preconditioning with salt, cold, or their combination enhances the survival of extremotolerant *A. pullulans* and *H. werneckii* under acute water-limiting stresses, specifically desiccation and freezing, using colony-forming units to measure viability. Additionally, we assessed the growth of both species across a salinity-temperature matrix to determine whether the effects of combined stressors are additive or act synergistically. The growth assays also re-evaluate the temperature and salt optima of both species, compare responses within and between species (e.g., generalists *A. pullulans* and *A. melanogenum* vs. specialists *A. subglaciale* and *H. werneckii*), and between *H. werneckii* strains of different ploidy.

## 2. Materials and Methods

### 2.1. Strains

All strains used in the study were acquired from and are stored in the Ex Culture Collection, part of the infrastructural center Mycosmo (MRIC UL, https://www.ex-genebank.com/index.php/en/), at the Department of Biology, Biotechnical Faculty, University of Ljubljana (Ljubljana, Slovenia). The selection included two strains of the generalist polyextremotolerant *A. pullulans* (EXF-150 and EXF-3645), one strain of a generalist but mostly aquatic species *A. melanogenum* (EXF-3378), one strain of the cold-environment specialist *A. subglaciale* (EXF-2481), two haploid strains of the halotolerant specialist *H. werneckii* (EXF-15 and EXF-562), and one diploid hybrid of the same species (EXF-2000) (Table 1). The strains were selected to represent ecological and physiological diversity within the *Aureobasidium* genus. For *H. werneckii*, genomic analyses indicate that the diploid genome of EXF-2000 is a hybrid of two haploid lineages, represented by EXF-15 and EXF-562, making this isolate the closest available hybrid counterpart to the two haploids [20]. This selection allowed comparison of growth responses across diverse ecological backgrounds and evaluation of whether ploidy might be linked to osmoadaptation.

### 2.2. Experimental Design

#### 2.2.1. Freezing and Desiccation Survival After Stress Preconditioning

The experiment aimed to determine whether prior exposure to low-temperature or high-salinity stress (or a combination of both) enhances fungal survival under freezing and desiccation. Two extremotolerant strains were used for these experiments: *A. pullulans* EXF-150 and *H. werneckii* EXF-2000.

Cultures were grown on YNB-Glc agar (0.17% yeast nitrogen base (YNB; Becton, Dickinson and Company, Franklin Lakes, NJ, USA), 0.5% (NH_4_)_2_SO_4_ (Sigma-Aldrich, St. Louis, MO, USA), 0.2% glucose (Kemika, Zagreb, Croatia), and 2% (*w*/*v*) agar (Biolife, Milan, Italy); pH 7) at 25 °C until visible colonies developed. A single colony was transferred to 20 mL of liquid YNB-Glc medium in 50 mL Falcon tubes to establish precultures, which were incubated overnight at 25 °C with shaking at 180 rpm. Precultures were used to inoculate 200 mL of fresh YNB-Glc medium in 500 mL Erlenmeyer flasks. The inoculation volume was calculated to achieve a starting optical density (OD_600_) of 0.05. Preconditioned cultures were incubated at 180 rpm until reaching mid-logarithmic phase (OD_600_ = 0.8–1.0) under one of the following four conditions:

1. Control: 25 °C in standard YNB-Glc medium
  (*A. pullulans* ~20 h; *H. werneckii* ~36 h)
2. Cold stress: 15 °C in standard YNB-Glc medium
  (*A. pullulans* ~2 d; *H. werneckii* ~4.5 d)
3. Salt stress: 25 °C in YNB-Glc medium supplemented with
  - 17% (*w*/*v*) NaCl (Chemlab, Zedelgem, Belgium) for *A. pullulans* (~14 d)
  - 25% (*w*/*v*) NaCl for *H. werneckii* (~20 d)
  (Adjusted to the upper halotolerance limit of each species)
4. Combined salt and cold stress: 15 °C in salt-supplemented YNB-Glc medium (as above; *A. pullulans* ~20 d; *H. werneckii* ~60 d)

Equal volumes of the cultures were harvested by vacuum filtration through 0.45 µm membrane filters (Merck, Burlington, MA, USA). Viability was measured by determining colony-forming units (CFU), before and after exposure to freezing and desiccation. For baseline viability, cells from one filter were resuspended in 0.9% NaCl, serially diluted, and plated on YNB-Glc agar. For freezing assays, the filter was stored at −20 °C for one or for seven days (to assess short- and long-term survival), thawed, and processed as above. For desiccation assays, the filter was dried in a desiccator with silica gel for one or for seven days at 25 °C, rehydrated in 0.9% NaCl, and processed as above. Plates were incubated at 25 °C for several days until colonies developed, and CFUs could be counted. All conditions were performed in three biological replicates for each fungus.

#### 2.2.2. Growth Assays over a Range of Salinities, Temperatures and Their Combinations

The experimental set included *H. werneckii* strains EXF-15 and EXF-562 (both haploid) and EXF-2000 (diploid), as well as *Aureobasidium* spp. strains EXF-150, EXF-3645, EXF-2481, and EXF-3378 (all haploid). The design enabled comparisons among strains of the same species, between species of the genus *Aureobasidium*, between the two genera, and between diploid and haploid strains of *H. werneckii*.

Growth experiments were performed using the Bioscreen C Pro system (Oy Growth Curves Ab Ltd., Helsinki, Finland), which enables continuous optical density (OD_600_) monitoring under controlled conditions. Each strain was tested in three biological replicates. Precultures were grown in liquid YNB-Glc medium (composition above) to OD_600_ = 1.0. For inoculation, 30 µL of culture was added to 270 µL of YNB-Glc medium with the appropriate NaCl concentration, yielding a starting OD_600_ of 0.1. For *Aureobasidium* spp., NaCl concentrations ranged from 0% to 17% (*w*/*v*) in 2% increments, with the upper limit chosen based on the reported halotolerance of *A. pullulans* [22]. For *H. werneckii*, NaCl concentrations ranged from 0% to 27.5% (*w*/*v*), with 5% increments at the lower end and 2.5% increments at the upper end to better capture changes near the halotolerance threshold. Media were prepared with a higher concentration of salt to account for dilution upon culture addition. The experiments were run at six temperatures (15, 20, 25, 30, 35, and 37 °C) with continuous orbital shaking at 180 rpm. Optical density (OD_600_) was measured every 30 min in a 14-day window.

### 2.3. Data Analysis

#### 2.3.1. Freezing and Desiccation Survival After Stress Preconditioning

For each stress treatment (desiccation and freezing), survival ratios of cultures grown under optimal conditions were compared with those of cultures preconditioned with high salinity, low temperature, or their combination. Survival ratios were calculated as the percentage of colony-forming units (CFUs) after stress exposure relative to the initial viable count. Statistical analyses were conducted in R (v4.5.1). Data were approximately symmetrically distributed with no extreme values, and group variances were comparable across treatments. One-way ANOVA was therefore applied, followed by Tukey’s HSD for pairwise comparisons. Additionally, due to the moderate number of data points, the non-parametric Kruskal–Wallis test with Dunn’s post hoc and Benjamini–Hochberg correction was performed and results compared with the results of ANOVA. Visualizations were generated in R using ggplot2 (v3.5.2) [72].

#### 2.3.2. Growth Assays over a Range of Salinities, Temperatures, and Their Combinations

OD_600_ data from the Bioscreen C Pro device were formatted per QurvE guidelines [73] and analyzed in R (v4.5.1) [74] with QurvE (v1.1.1) [73]. For each replicate, growth curves were fitted, and the generation time was extracted. Mean doubling times and standard deviations were computed for each combination of strain, temperature, and salinity. Matrix plots were produced in R with ggplot2 (v3.5.2) [72].

To evaluate how salinity and temperature influenced fungal growth, doubling times were log-transformed. A common set of linear models was applied to pooled *Aureobasidium* spp. and *H. werneckii* data to determine whether their overall growth responses were best explained by salinity, temperature, their additive effects, or their interaction. The candidate models included: (i) the null model (intercept-only), (ii) only NaCl as independent variable, (iii) only temperature as independent variable, (iv) the additive term (NaCl + Temperature), and (v) the interaction term (NaCl × Temperature). Model performance was compared using Akaike’s Information Criterion (AIC) [75], with models considered equally supported when the difference in AIC (ΔAIC) was ≤2. When combinations of salinity and temperature were too extreme to support growth, doubling times could not be estimated. Therefore, linear models were fit only to the conditions for which growth data were available.

For *Aureobasidium* spp. (*A. pullulans*, *A. subglaciale*, *A. melanogenum*), once the NaCl × Temperature interaction was identified as the best-supported model, it was extended to include species identity (NaCl × Temperature × Species). This extension made it possible to test whether the magnitude and direction of salinity-temperature interactions varied among species. The extended model allowed each species to have its own unique baseline growth rate and distinct response patterns to salinity and temperature. Species-specific slopes for salinity and temperature were estimated using the emmeans package [76] in R (v4.5.1) [74], providing species-specific estimates of how doubling times change with each environmental variable. As a robustness check and to examine intraspecific variation, the entire set of core models was also fit separately for each species and each individual strain. This approach helped validate species-level patterns and identify potential sources of strain-level heterogeneity in responses to salinity and temperature.

For *H. werneckii*, the same set of core models was applied to pooled strain data. In the next step ploidy (haploid vs. diploid) was incorporated as a grouping factor, to assess its influence on growth responses. Two of the best-supported core models were extended to include ploidy as a full interaction term (NaCl × Ploidy; ((NaCl + Temperature) × Ploidy), allowing haploid and diploid strains to differ in both baseline doubling times and their responses to stress factors. The three-way interaction model (Ploidy × NaCl × Temperature) was added to test whether the combined effects of salinity and temperature on growth changed with ploidy. That is, whether the interaction between salinity and temperature affects growth differs between haploid and diploid strains. This model thus provided a framework to evaluate whether multiple stress factors exert additive or synergistic effects across different ploidy states. Strain-specific slopes for salinity effects were extracted from the best-supported model (NaCl × Ploidy) using the emmeans package (v1.11.2) [76], providing separate estimates for haploid and diploid strains. To investigate potential temperature effects that may have been masked by strain-level variation, the full set of core models was also applied individually to each *H. werneckii* strain. All analyses were performed in the R environment [74].

## 3. Results

### 3.1. Freezing and Desiccation Survival After Stress Preconditioning

We tested whether preconditioning with salt (NaCl), cold, or their combination affected desiccation and freezing survival of *Aureobasidium pullulans* EXF-150 and *Hortaea werneckii* EXF-2000.

**Figure 1:**
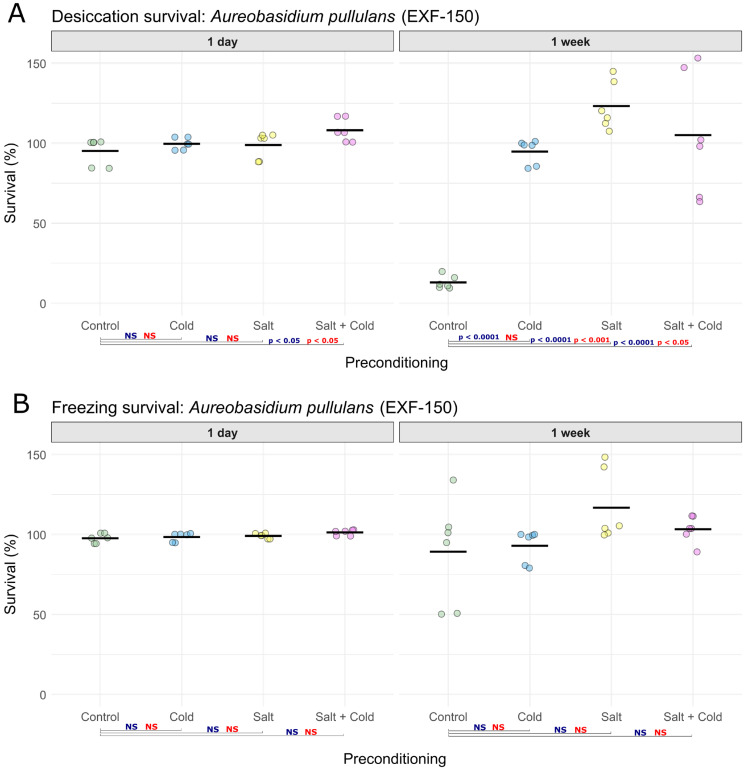
Survival of *Aureobasidium pullulans* EXF-150 following preconditioning under different stress conditions. (**A**) Desiccation survival and (**B**) freezing survival, each measured after 1 day and 1 week of exposure (*n* = 6 per condition). Survival is expressed as the percentage of colony-forming units (CFUs) remaining relative to the initial viable count before exposure to freezing or desiccation. Preconditioning treatments included: control (25 °C), cold (15 °C), salt (17% NaCl, 25 °C), and combined salt and cold (17% NaCl, 15 °C). Colored circles represent individual replicates within each of the tested conditions. Control samples are shown in green, cold-preconditioned samples in blue, salt-preconditioned samples in yellow, and combined salt and cold-preconditioned samples in pink. Horizontal black lines indicate mean survival values for each condition. Blue labels indicate *p*-value thresholds from Tukey’s test, red labels indicate *p*-value thresholds from Dunn’s test, and “NS”, color-coded accordingly, denotes non-significant comparisons.

In *A. pullulans*, desiccation survival after one day remained high across all treatments (*n* = 6 per condition) (ANOVA: F(3,20) = 3.55, *p* = 0.0330) (Figure 1A, Table S1). Tukey’s test indicated that only the combined NaCl and cold preconditioning increased survival relative to the control (*p* = 0.0239), which was also supported by the Kruskal–Wallis test (H(3) = 8.86, *p* = 0.0311) and Dunn’s testing (BH-adjusted *p* = 0.0288). After one week of desiccation (Figure 1A, Table S2), survival also differed significantly among treatments (*n* = 6 per condition, ANOVA: F(3,20) = 32.20, *p* < 0.0001). Compared with the control, survival increased after NaCl preconditioning (Tukey *p* < 0.0001), combined NaCl and cold (Tukey *p* < 0.0001), and cold preconditioning (Tukey *p* < 0.0001). Kruskal–Wallis test also detected a treatment effect (H(3) = 16.60, *p* = 0.0009), and Dunn’s tests identified significant effects of NaCl (BH-adjusted *p* = 0.0005) and combined NaCl and cold preconditioning (BH-adjusted *p* = 0.0120), whereas the effect of cold preconditioning was not significant (BH-adjusted *p* = 0.0864). By comparison, freezing survival remained close to 100% across all treatments, including in the control (*n* = 6 per condition; Figure 1B, Tables S1 and S2). Therefore, no significant differences were detected after one day (ANOVA: F(3,20) = 2.71, *p* = 0.0721) or after one week (ANOVA: F(3,20) = 2.08, *p* = 0.1360). The non-parametric Kruskal–Wallis tests supported the same conclusion (1 day: H(3) = 5.47, *p* = 0.1400; 1 week: H(3) = 7.55, *p* = 0.0562).

**Figure 2:**
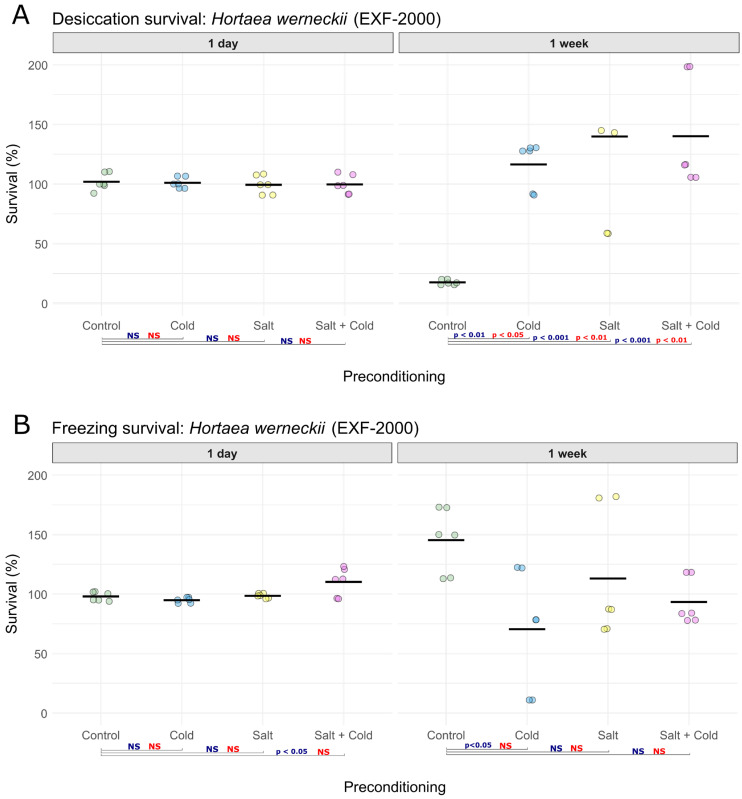
Survival of *Hortaea werneckii* EXF-2000 following preconditioning under different stress conditions. (**A**) Desiccation survival and (**B**) freezing survival, each measured after 1 day and 1 week of exposure (*n* = 6 per condition). Survival is expressed as the percentage of colony-forming units (CFUs) remaining relative to the initial viable count before exposure to freezing or desiccation. Preconditioning treatments included: control (25 °C), cold (15 °C), salt (25% NaCl, 25 °C), and combined salt and cold (25% NaCl, 15 °C). Colored circles represent individual replicates within each of the tested conditions. Control samples are shown in green, cold-preconditioned samples in blue, salt-preconditioned samples in yellow, and combined salt and cold-preconditioned samples in pink. Horizontal black lines indicate mean survival values for each condition. Blue labels indicate *p*-value thresholds from Tukey’s test, red labels indicate *p*-value thresholds from Dunn’s test, and “NS”, color-coded accordingly, denotes non-significant comparisons.

In *H. werneckii*, desiccation survival after one day remained approximately 100% across all treatments (*n* = 6 per condition; Figure 2A, Table S3), with no significant differences among treatments (ANOVA: F(3,20) = 0.18, *p* = 0.9100; Kruskal–Wallis: H(3) = 1.32, *p* = 0.7240). However, after one week (Figure 2A, Table S4), survival differed significantly between treatments (*n* = 6; ANOVA: F(3,20) = 10.91, *p* = 0.0002; Kruskal–Wallis: H(3) = 13.40, *p* = 0.0038). Tukey’s and Dunn’s post hoc tests indicated that all three preconditioning treatments increased survival relative to the control: cold (Tukey *p* = 0.0040; Dunn BH-adjusted *p* = 0.0179), NaCl (Tukey *p* = 0.0005; Dunn BH-adjusted *p* = 0.0065), and combined NaCl and cold (Tukey *p* = 0.0005; Dunn BH-adjusted *p* = 0.0098). After one day of freezing (Figure 2B, Table S3), survival differed among treatments (*n* = 6; ANOVA: F(3,20) = 6.86, *p* = 0.0023), with the combined NaCl and cold preconditioning resulting in higher survival than the control (Tukey *p* = 0.0152). However, this result should be interpreted with caution, as the observed difference was small and did not reach statistical significance in the non-parametric analysis (Kruskal–Wallis, H(3) = 8.24, *p* = 0.0414; Dunn’s test BH-adjusted *p* = 0.1960). After one week of freezing (Figure 2B, Table S4), ANOVA detected a treatment effect (*n* = 6; F(3,20) = 3.79, *p* = 0.0266), and Tukey’s post hoc test (*p* = 0.0199) showed a significant negative effect of the cold preconditioning of freezing. The non-parametric Kruskal–Wallis test detected no impact of preconditioning (H(3) = 6.80, *p* = 0.0785).

### 3.2. Growth Assays over a Range of Salinities, Temperatures, and Their Combinations

**Figure 3:**
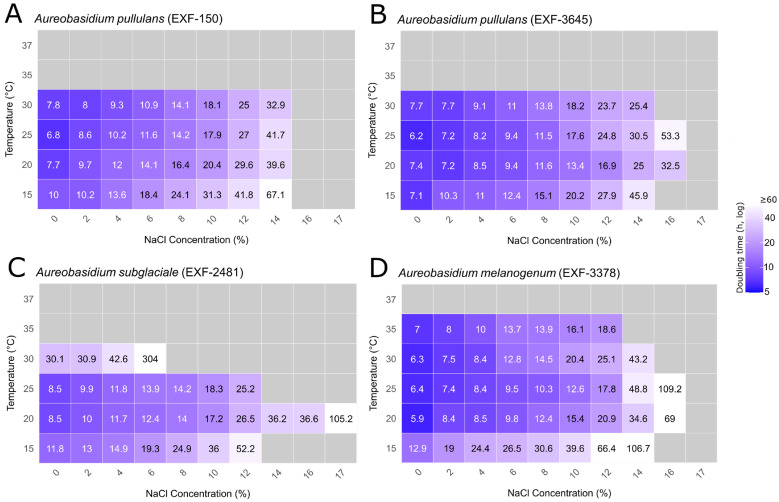
Heatmaps of mean generation times for *Aureobasidium* spp. across salinity and temperature gradients. Panels show: (**A**) *A. pullulans* EXF-150, (**B**) *A. pullulans* EXF-3645, (**C**) *A. subglaciale* EXF-2481, and (**D**) *A. melanogenum* EXF-3378. Numbers within tiles represent mean generation times (in hours) across biological replicates. Colour shading corresponds to mean generation time, with darker blue shades indicating faster growth. Grey tiles indicate tested conditions where no growth was detected.

**Figure 4:**
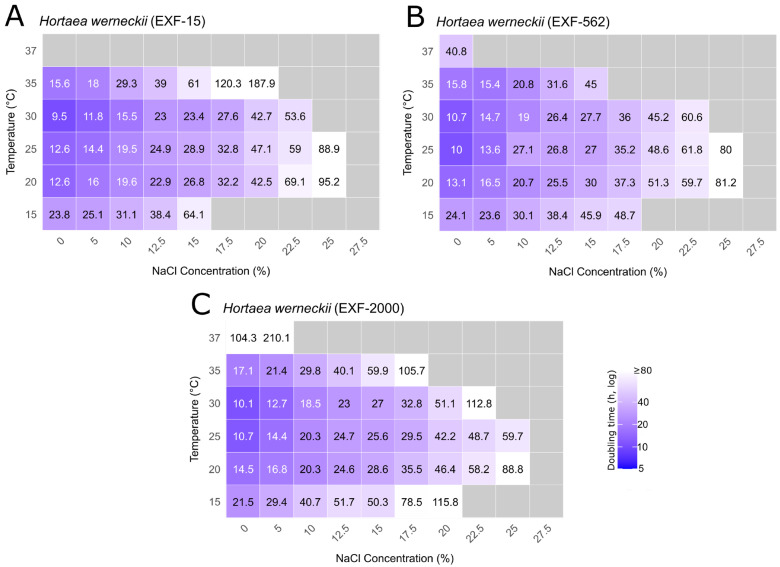
Heatmaps of mean generation times for *Hortaea werneckii* across salinity and temperature gradients. Panels show: (**A**) EXF-15, (**B**) EXF-562, and (**C**) EXF-2000. Numbers within tiles indicate mean generation times (in hours) across biological replicates. Colour shading corresponds to mean generation time, with darker blue shades indicating faster growth. Grey tiles indicate tested conditions where no growth was detected.

The experimental set included *H. werneckii* strains EXF-15 and EXF-562 (haploid) and EXF-2000 (diploid), as well as four *Aureobasidium* strains representing three species: *A. pullulans* EXF-150 and EXF-3645, *A. subglaciale* EXF-2481, and *A. melanogenum* EXF-3378. The resulting heatmaps (Figure 3 and Figure 4; Tables S5–S11) display mean generation times for each strain across all tested salinity (NaCl) and temperature combinations. All *Aureobasidium* spp. and *H. werneckii* strains exhibited measurable growth at moderate salt concentrations and temperatures, with no growth indicated by grey tiles.

Across salinity and temperature gradients, *Aureobasidium* strains exhibited species- and strain-specific growth patterns (Figure 3), suggesting evolutionary adaptation to the prevailing conditions of their native environments. None of the tested *Aureobasidium* strains grew at 37 °C, and growth was generally reduced at temperatures deviating from the thermal optimum and at higher salinities.

*A. pullulans* EXF-150 grew across a broad salinity range (0–14% NaCl) at most temperatures, with fastest growth occurring between 20 °C and 30 °C in a non-saline medium, where doubling time ranged from 7 to 8 h, indicating a preference for moderate temperatures and minimal salinity (Figure 3A; Table S5). *A. pullulans* EXF-3645 behaved similarly but grew faster overall and tolerated salt better than EXF-150. Growth occurred from 0% to 14% NaCl at temperatures between 15 °C and 30 °C; however, like EXF-150, it failed to grow at 35 °C. Notably, EXF-3645 sustained growth at 16% NaCl at 20 °C and 25 °C, conditions that completely inhibited EXF-150, highlighting its superior salt tolerance (Figure 3B, Table S6).

*A. subglaciale* EXF-2481 was the slowest-growing *Aureobasidium* strain, with the narrowest salt tolerance range. It grew between 0% and 12% NaCl, but only between 15 °C and 25 °C, with no growth detected at 35 °C. Optimal growth, with doubling times of 8–10 h, was observed at 20–25 °C under low-salt conditions. At 30 °C and 6% NaCl, doubling times exceeded 300 h, or growth ceased entirely. Interestingly, this was the only strain that grew at 17% NaCl, though exclusively at 20 °C, suggesting that this temperature represents its physiological optimum where halotolerance capacity is maximized (Figure 3C, Table S7).

*A. melanogenum* EXF-3378 exhibited the broadest temperature tolerance among all tested *Aureobasidium* strains, growing across 0–14% NaCl at temperatures ranging from 15 to 30 °C. The fastest growth, with doubling times of 6–7 h, was observed at 30–35 °C and 0–2% NaCl. Notably, *A. melanogenum* maintained measurable growth at 35 °C even under moderate salt stress (up to 12% NaCl), demonstrating superior thermotolerance compared to the other *Aureobasidium* strains in this study (Figure 3D, Table S8).

The growth of *H. werneckii* strains across salinity and temperature gradients revealed distinct physiological profiles among the three isolates (Figure 4, Tables S9–S11). Across all tested strains, growth was slower at temperatures outside the optimal range and generally declined with increasing salinity, indicating a reduced performance under stress.

EXF-15 grew across a wide salinity range (0–25% NaCl), although growth at the highest salinity was detected only at 20 °C and 25 °C. At lower salinities, the strain grew between 15 °C and 35 °C, with the fastest growth (doubling times of 9.5–12.6 h) observed between 20 °C and 30 °C and 0–5% NaCl. At 37 °C, no growth was detected under any salinity condition (Figure 4A, Table S9).

EXF-562 showed a comparable salt tolerance, with growth at 25% NaCl observed at 20 °C and 25 °C (doubling times of 81.2 and 80 h, respectively). Unlike EXF-15, this strain also grew at 37 °C under non-saline conditions, albeit slowly (doubling time of 40.8 h). Optimal growth (10–16.5 h) was recorded at 20–30 °C and 0–5% NaCl (Figure 4B, Table S10).

EXF-2000 grew in the range of 0% to 25% NaCl, with the fastest growth at 20–30 °C and 0–5% NaCl (doubling times of 10.1–14.4 h). At these temperatures, growth remained relatively stable up to 12.5% NaCl before declining sharply at higher concentrations. At 35 °C, growth persisted up to 17.5% NaCl, and at 37 °C, limited growth was still detectable at 0% and 5% NaCl (doubling times of 104.3 and 210.1 h), indicating that this diploid strain tolerated elevated temperatures better, but with substantially reduced growth efficiency (Figure 4C, Table S11).

Growth responses to salinity and temperature were analyzed using linear models to identify the main environmental drivers of variation in fungal doubling times. For *Aureobasidium* spp., we first evaluated a set of core linear models to test whether variation in doubling times was better explained by salinity, temperature, their additive effect, or their interaction (Table 2). Model comparison based on AIC indicated that the interaction model including NaCl × Temperature provided the best fit to the data (AIC = 508.93, weight = 9.69 × 10^−30^). The additive model was the next best (AIC = 517.32, ΔAIC = 8.39, weight = 1.46 × 10^−31^), while models including only salinity (AIC = 533.25, weight = 5.08 × 10^−35^) or only temperature (AIC = 848.82, weight = 1.51 × 10^−103^) performed substantially worse. The null model was least supported (AIC = 860.66, weight = 4.06 × 10^−106^). Thus, the growth of *Aureobasidium* spp. was jointly influenced by salinity and temperature, and their interaction. The model was refined to include species identity, allowing evaluation of species-specific responses to the combined effects of salinity and temperature (NaCl × Temperature × Species). This model was strongly supported (AIC = 375.32, weight = 1) compared with the simpler NaCl × Temperature model (AIC = 508.93, ΔAIC = 133.61, weight = 9.69 × 10^−30^), indicating that including the species variable greatly improved model performance and revealed pronounced interspecific differences in how the interaction of salinity and temperature affected growth. This shows that both the baseline growth (intercept) and the strength or direction of the salinity-temperature interaction varied among species. Estimated marginal trends showed that increasing salinity consistently slowed growth in all three *Aureobasidium* species. The strongest effect was observed in *A. melanogenum*, where on average, a 1% increase in NaCl prolonged doubling time by 0.126 h (*p* < 0.0001). *A. pullulans* also showed a pronounced effect (0.112 h per 1% NaCl, *p* < 0.0001), while *A. subglaciale* was least affected (0.075 h per 1% NaCl, *p* < 0.0001). Based on a comparison of their respective doubling time prolongation rates per 1% NaCl increase, the salinity sensitivity of *A. melanogenum* was 68% greater than that of *A. subglaciale*.

Temperature effects diverged more markedly among species. Within the permissive growth range, higher temperatures were associated with shorter doubling times in *A. melanogenum* (−0.035 h per 1 °C, *p* < 0.0001) and *A. pullulans* (−0.019 h per 1 °C, *p* = 0.0001), whereas *A. subglaciale* showed the opposite, with doubling times increasing slightly at higher temperatures (+0.034 h per 1 °C, *p* = 0.0009). These contrasting responses indicate that *A. subglaciale* is better adapted to cooler conditions, while *A. melanogenum* and *A. pullulans* are better adapted to warmer environments. Overall, the slope estimates demonstrate clear and biologically meaningful differences in both salinity sensitivity and thermal strategy among species.

Strain-level core models were also tested as a robustness check to assess whether species-level pooling was justified (Table S12). The main patterns observed at the species level were generally consistent, as interactions between salinity and temperature were present in *A. pullulans* EXF-150 and *A. subglaciale* EXF-2481. In contrast, *A. melanogenum* EXF-3378 and *A. pullulans* EXF-3645 provided equivocal support (ΔAIC < 2), so interaction and additive models could not be distinguished from each other.

In summary, salinity reduced growth in all *Aureobasidium* species, although the strength of this effect varied, with *A. melanogenum* being the most sensitive. Temperature responses differed qualitatively: *A. melanogenum* and *A. pullulans* grew faster at warmer temperatures, whereas *A. subglaciale* slowed down, demonstrating significant interspecific variation in thermal strategies among closely related species. Growth collapsed entirely at extreme salinity or temperature levels, where doubling times could not be estimated; linear models were therefore fitted only to conditions where measurable growth occurred.

For *H. werneckii*, core model comparisons across all strains indicated that variation in doubling times was best explained by the additive effect of salinity and temperature, and by salinity alone (Table 3). The NaCl-only model provided the best fit (AIC = 626.86, weight = 3.02 × 10^−25^), while the additive model including temperature differed by two AIC units (AIC = 628.85, ΔAIC = 1.99, weight = 1.11 × 10^−25^). The interaction model (NaCl × Temperature) (AIC = 630.8, weight = 4.21 × 10^−26^) performed worse than the additive model. The temperature-only model performed worse than the null model (AIC = 689.38; weight = 8.01 × 10^−39^ vs. 687.47; weight = 2.08 × 10^−38^), confirming that temperature alone has no explanatory power for growth variation when strains are pooled. Extending the NaCl-only model and additive models with ploidy as a full interaction substantially improved fit, with the NaCl × ploidy model being better suited (AIC = 514.36; weight = 8.09 × 10^−1^ vs. 517.65; weight = 1.57 × 10^−1^), indicating that the data are best explained by the NaCl-only model, and that haploid and diploid strains differ in their salinity responses. Adding a three-way interaction model (Ploidy × NaCl × Temperature) allowed for testing whether the combined effects of salinity and temperature on growth depended on ploidy. However, this model (AIC = 520.17, weight = 3.38 × 10^−2^) did not improve fit compared to simpler models (ΔAIC = 6.35 relative to NaCl × ploidy), suggesting that haploid and diploid strains respond similarly to simultaneous salinity and temperature stress.

Analysis of the linear coefficients using the best-fitting NaCl × ploidy interaction model revealed ploidy-specific responses. For haploid strains, each 1% increase in NaCl prolonged doubling time by 0.067 h (*p* < 0.0001), reflecting strong salinity sensitivity. The diploid strain showed a contrasting pattern with a slope estimate close to zero (−0.021 h per 1% NaCl) and not significant (*p* = 0.7083), indicating no detectable effect of salinity on doubling time in the diploid strain.

Strain-level models revealed additional details not captured in the pooled analysis (Table S12). In EXF-15 (haploid), the interaction model (NaCl × Temperature) provided the best fit (AIC = 104.16, weight = 9.73 × 10^−1^), substantially outperforming the NaCl-only model (AIC = 111.96, ΔAIC = 7.80, weight = 1.97 × 10^−2^) and the additive model (AIC = 113.89, ΔAIC = 9.73, weight = 7.50 × 10^−3^). In EXF-562 (haploid), the interaction model was also best supported (AIC = 32.72, weight = 6.30 × 10^−1^), but the NaCl-only and additive models were relatively close in terms of model support to the best model (ΔAIC = 2.09 and 2.88, respectively). In EXF-2000 (diploid), the interaction model again provided the best fit (AIC = 213.06, weight = 9.71 × 10^−1^), followed by the additive model (AIC = 220.67, ΔAIC = 7.61, weight = 2.16 × 10^−2^). The NaCl-only model was substantially worse (AIC = 222.90, ΔAIC = 9.84, weight = 7.08 × 10^−3^).

In summary, salinity dominated growth responses in *H. werneckii*, with haploid strains highly sensitive to salt and the diploid strain showing no detectable salinity effect when pooled. At the pooled level, temperature contributed little explanatory power. Strain-level analyses revealed that EXF-15 (haploid) and EXF-2000 (diploid) both exhibited clear NaCl × temperature interactions, while EXF-562 (haploid) produced ambiguous results with the NaCl-only model, while the additive and the interaction models were statistically indistinguishable. These findings highlight ploidy-linked differences in salinity responses and show that pooling can obscure important strain-specific effects of temperature.

## 4. Discussion

Organisms that persist in extreme environments are rarely exposed to single stressors in isolation. Instead, survival depends on the ability to withstand combinations of overlapping stresses whose joint impact can exceed simple additive effects. Nevertheless, many studies of stress tolerance continue to focus on stress factors applied individually [21,40,47,77]. This approach risks underestimating both the magnitude and the ecological relevance of stress responses, particularly in extremotolerant organisms. This issue is especially pertinent in extremotolerant fungi, whose long-term persistence may rely either on flexible, inducible responses or on constitutive preparedness shaped by chronic exposure to harsh conditions. Understanding how these contrasting strategies influence survival under acute stress remains a central challenge in stress biology. To address this knowledge gap, we first investigated whether preconditioning with salt, cold, or their combination enhances the survival of *Aureobasidium pullulans* and *Hortaea werneckii* under acute water-limiting stresses (specifically desiccation and freezing).

Our results show that preconditioning can enhance desiccation survival in both species, particularly under prolonged exposure. However, the magnitude and the level of statistical support differed between species, reflecting their fundamentally different survival strategies [1]. *Aureobasidium pullulans* acted as a stress-tolerant generalist in which preconditioning triggered large changes in desiccation survival. *Hortaea werneckii* relied on a more specialized strategy characterized by broadly constitutive stress protection and a reduced reliance on inducible responses.

In *A. pullulans*, all three preconditioning regimes increased long-term desiccation survival, although the effect of cold alone was less pronounced and offered only a limited protection, whereas salt-based treatments induced a more effective adaptive response. Preconditioning effects were weaker after one day of desiccation, where only the combined salt and cold treatment resulted in higher mean survival, likely because the shorter treatment was less detrimental, and survival remained high even in the control. These observations are consistent with *A. pullulans* being a phenotypically plastic generalist species capable of entering anhydrobiosis under severe water stress [21], and employing a bet-hedging strategy, in which diverse physiological and morphological states buffer environmental variability [13,17]. This interpretation also aligns with genomic evidence showing frequent recombination and high intraspecific diversity in *A. pullulans*, supporting its capacity for ecological plasticity with no signs of intraspecific specialization [12,13]. Such inducible tolerance may also help explain the wide biotechnological utility of the species in biocontrol, fermentation, and material production [19,32].

In *H. werneckii*, all preconditioning treatments improved one-week desiccation survival (although with higher *p*-values than in *A. pullulans*), indicating a generally positive impact of preconditioning. No significant effects of preconditioning on short-term desiccation survival were observed in this species. *H. werneckii* is a stress specialist, highly adapted to persistently hypersaline environments such as hypersaline salterns and salt-saturated surfaces [14,39,40,43]. Genomic and transcriptomic studies indicate that *H. werneckii* constitutively expresses a broad suite of stress-related genes, including osmoprotectants, ion transporters, and antioxidative enzymes [16,44,51,54,56,57]. Its stress tolerance is therefore less flexible and primed for osmotic and desiccation-like challenges without requiring further induction. Future transcriptomic or proteomic studies will help clarify to what extent these survival traits are adaptable and to what extent they are hardwired.

Salt and cold stresses activate overlapping protective pathways in fungi, including the accumulation of compatible solutes, ion regulation, cell wall remodelling, changes in membrane composition, and induction of stress-protective proteins and antioxidant defences [1,14,15,18,21,47,58,61]. Because these responses are shared, they are difficult to disentangle. Nevertheless, our data suggest that NaCl preconditioning more effectively primes fungi for desiccation survival, whereas cold alone provides limited benefits. This may reflect a closer physiological similarity between osmotic stress and desiccation, both of which involve cellular dehydration and solute accumulation. Taken together, these findings both reinforce established concepts of stress adaptation in extremotolerant fungi and highlight species-specific divergences that reflect contrasting ecological strategies and modes of stress preparedness. While desiccation strongly affected the viability of the cultures, freezing survival generally remained high (approximately 100%) in both control and preconditioned cultures of both fungi. Notably, a modest reduction in survival after one week of freezing following cold preconditioning was observed in *H. werneckii*, although this effect was found to be significant only by the parametric analysis, and should therefore be interpreted with caution. Because the freezing was not detectably detrimental to survival after both one day and one week of freezing at −20 °C, the impact of preconditioning could not be meaningfully tested. This was unexpected, as freezing without external cryoprotectants or flash-freezing in liquid nitrogen should cause substantial cellular damage due to ice crystal formation [21]. This intrinsic tolerance to freezing of both species suggest that keeping them at −20 °C may be a viable short- to medium-term storage possibility, even though such conditions would normally be considered entirely suboptimal for such purpose. In terms of viability studies during freezing, longer freezing periods or multiple freeze–thaw cycles should be used in the future.

It should be noted that preconditioning duration differed between treatments, as cultures were harvested at comparable physiological states, specifically mid-logarithmic phase of growth, rather than after equal incubation times, because increasingly stringent stress conditions required longer incubation periods for cultures to reach the desired optical density, which may have influenced the magnitude of the observed effects. Interestingly, we observed that in some cases the number of CFU after stress exposure increased above that of the initial culture before stress exposure. This effect, most pronounced after one week of desiccation or freezing, may reflect the resuscitation of previously non-culturable cells, phenotypic heterogeneity leading to replicate-specific outgrowth, the disintegration of multicellular CFUs, or simply technical variation in plating.

In addition to survival under acute stress, we examined growth responses of black yeasts to combined gradients of salinity and temperature. Examining fungal growth across these gradients provides insight into whether salinity and temperature act additively or synergistically to shape growth. The study also enabled a re-evaluation of strain-specific temperature and salinity optima and facilitated comparisons of responses within and between species as well as between *Aureobasidium* spp. and *H. werneckii*. This analysis encompassed seven strains representing four species, including *A. pullulans* (EXF-150, EXF-3645), *A. subglaciale* (EXF-2481), *A. melanogenum* (EXF-3378), and *H. werneckii* (EXF-15, EXF-562, EXF-2000), the latter including both haploid and diploid lineages to evaluate the influence of ploidy on growth responses.

In *Aureobasidium* spp., prolongation of doubling times was best explained by a synergistic effect of temperature and salinity, rather than by an independent effect of either factor. The strength of this interaction varied systematically among species, consistent with differences in ecological strategy. Salinity inhibited growth in all species, but sensitivity followed a clear gradient, with *A. melanogenum* being the most affected, *A. pullulans* intermediate, and *A. subglaciale* the least affected species. Correspondingly, thermal preferences differed, with *A. subglaciale* favouring lower temperatures, *A. melanogenum* higher temperatures, and *A. pullulans* occupying an intermediate position. These species-level patterns align with their known ecological strategies: *A. pullulans* is a broad generalist [1,13,22], *A. melanogenum* is moderately specialized and warm-adapted [12,21,22], while *A. subglaciale* is a cold-adapted specialist [12,21,22,35]. This thermal differentiation also reflects distinct climatic zones from which these species were originally isolated. When analyzed separately, strain-level models largely mirrored the species-level results, although the magnitude of the temperature–salinity synergy varied among genotypes. Stronger synergistic interactions were evident in *A. pullulans* EXF-150 and *A. subglaciale* EXF-2481, whereas *A. melanogenum* EXF-3378 and *A. pullulans* EXF-3645 exhibited weaker, near-additive responses. As seen in the viability experiments discussed above, variability in growth responses also reflects a high degree of physiological flexibility, suggesting that *Aureobasidium* species can dynamically modulate their stress responses depending on environmental context.

In *H. werneckii*, variation in growth responses was primarily driven by salinity rather than temperature, consistent with its extreme halotolerance. Haploid strains exhibited a clear and significant prolongation of doubling time with increasing NaCl concentration, whereas the diploid strain displayed an attenuated response, with salinity still prolonging doubling time but to a much lesser extent. This pattern suggests a buffering effect of genome duplication, either through gene dosage effects, heterozygosity at key loci or other effects. Notably, the combined influence of temperature and salinity was not modulated by ploidy, indicating that genome duplication primarily mitigates osmotic stress rather than altering temperature sensitivity. The large number of diploid *H. werneckii* strains isolated from nature and their unusual origin in several independent hybridizations of highly heterozygous parents despite the clonal nature of the species [20] has long been suggested as a possible adaptation to hypersaline conditions [20,54,78]. Nevertheless, this is the first clear evidence supporting such a hypothesis. At the strain level, both haploid (EXF-15 and EXF-562) and diploid (EXF-2000) strains indicated that salinity and temperature act jointly to shape growth, although the strength of this interaction differed among strains. EXF-15 and EXF-2000 showed clear temperature-dependent effects of salinity, whereas EXF-562 exhibited a weaker or near-additive response. When all strains were analyzed together, temperature had no explanatory power, indicating that its influence emerges primarily through a synergistic interaction with salinity rather than as an independent factor. Importantly, this also illustrates a form of Simpson’s paradox, where pooled analyses obscure or even reverse trends that become evident only when data are examined at the strain level. Biologically, these results highlight that while *H. werneckii*’s growth is dominated by osmotic regulation; thermal effects emerge only contextually under salt stress. This supports the view that *H. werneckii* is a specialized halotolerant species shaped by constant hypersaline environments.

For many strains of *Aureobasidium* spp. and *Hortaea werneckii*, precise temperature and salinity optima are still poorly defined in the literature. Research on these extremotolerant fungi has focused mainly on tolerance limits rather than systematic characterization of growth performance across environmental gradients, even of a single, let alone a combination of environmental factors.

Based on the literature, *A. pullulans* exhibits optimal growth at 20–30 °C [79,80] with a preference for low salinity conditions [14] and a growth limit at 17% NaCl (*w*/*v*) [21]. This is consistent with our observations for strains EXF-150 and EXF-3645, which reached their fastest growth at 20–30 °C and 0–2% NaCl, while the maximum salinity limit was reached at 14% (EXF-150) and 16% (EXF-3645) NaCl.

*A. subglaciale* is characterized by psychrophilic tendencies (it grows at 4 °C) [12,22], and has been reported to tolerate up to 10% NaCl [21]. This aligns with the performance of the strain EXF-2481 in this study. It exhibited optimal growth between 20 °C and 25 °C, and at low salinities (0–2% NaCl). However, it was also able to grow at 17% NaCl, far exceeding the previously reported salinity limit for *A. subglaciale* [22]. The difference may be due to testing typically being performed at 24 °C and on solid media. Here, the unexpected halotolerance of *A. subglaciale* was observed at 20 °C, illustrating the impact that a one-size-fits-all testing approach can have on results.

*A. melanogenum* is known to have a higher temperature optimum than *A. pullulans* and to be capable of growth at 37 °C [1,21]. This is somewhat consistent with the behaviour of the strain EXF-3378 in this study. It exhibited a broad temperature range of 20–35 °C under low-salinity conditions and tolerated up to 16% NaCl (*w*/*v*). However, no growth was observed at 37 °C. Again, the difference may stem from differences in the testing methods (e.g., growth on solid vs. in liquid medium).

For *H. werneckii*, the literature describes an optimal salinity range of 6–10% NaCl (or 0.8–1.7 M) and an optimal growth temperature around 25 °C, whereas the three strains included in our study (EXF-15, EXF-562, EXF-2000) achieved their fastest growth at lower salinities (0–5% NaCl) and within a broader temperature range of 20–30 °C. Previous reports note that growth ceases close to NaCl saturation (e.g., 30% NaCl) [81], but in our dataset, all three strains were only able to grow at salinities up to 25% NaCl.

The reported observations clearly illustrate why the impact of the chosen methodology on the determined growth optima and limits should not be underestimated, and no method is without its own shortcomings. While the Bioscreen C Pro system is highly effective for precise determination of growth curves under different conditions and excels in determining the growth optima, it has several limitations in defining the absolute ceiling of extremotolerance. The 14-day experimental window, constrained by factors such as medium evaporation and morphological transitions, may result in underestimated tolerance range. The long adaptation period or slow growth may exceed the two-week window rather than indicate death or inability to reproduce. The “no growth” designations in this study are in several cases actually extended lag phases near the extremes, which could not be used to determine the doubling times. The evaporation of the medium results in a higher concentration of salt than the starting concentration. The defensive shifts in morphology from single cells to clumped, aggregated forms and hyphae directly interfere with the light-based OD measurement, especially in the phenotypically very plastic black yeasts. To better capture growth near physiological limits, alternative approaches could include extended incubation on solid media, microcolony tracking or time-lapse microscopy, continuous cultivation in chemostat or turbidostat systems, and the use of sealed or gas-permeable microplates for prolonged incubation.

## 5. Conclusions

This study presents an integrated perspective on how extremotolerant black yeasts respond to multiple environmental stressors, clearly distinguishing two contrasting evolutionary strategies. Within *Aureobasidium*, species vary along a generalist-specialist continuum but collectively exhibit inducible and flexible stress responses, with growth determined by synergistic interactions between salinity and temperature that surpass simple additive effects. This flexibility also reflects the ecological preferences of individual species, as shown by the divergent thermal adaptations of *A. melanogenum* and *A. pullulans*, in which growth accelerates with increasing temperature, compared to *A. subglaciale*, in which the opposite is observed. In contrast, *H. werneckii* functions as a halotolerant specialist, with growth responses primarily influenced by salinity and minimal independent temperature effects. Notably, we found that halotolerance of *H. werneckii* is also modulated by ploidy: haploid strains were more impacted by salinity than the diploid strain, indicating that a hybrid genome of many *H. werneckii* strains buffers against osmotic stress.

Our results indicate an evolutionary trade-off of generalist vs. specialist strategies in adaptation to extreme conditions. While specialists such as *H. werneckii* sacrifice flexibility and responsiveness to novel or changing conditions, they maintain a consistently high level of preparedness for the stresses typical of their niche. In contrast, generalists such as *A. pullulans* remain agile and can quickly adapt to large environmental fluctuations [13]. The differences are reflected in a much wider distribution of *A. pullulans* compared to *H. werneckii*. The inducible tolerance in *A. pullulans* may be exploited for adaptive pre-treatment strategies in biocontrol or material production, whereas the robust performance of *H. werneckii* could be advantageous in high-salinity industrial processes.

Future research should build on these physiological insights by incorporating transcriptomic and proteomic analyses to elucidate the molecular mechanisms of constitutive versus dynamic regulation of extremotolerance. Comparative research across other extremotolerant fungi may reveal broader evolutionary patterns in the balance between flexible generalists and rigid specialists.

## Figures and Tables

**Table 1:** Fungal strains used in this study with their ecological origins.

Strain	Species	Isolation Source	Location
EXF-150	*Aureobasidium pullulans*	Hypersaline water, solar saltern	Sečovlje, Slovenia
EXF-3645	*Aureobasidium pullulans*	Glacial ice	Ny-Ålesund, Svalbard, Norway
EXF-2481	*Aureobasidium subglaciale*	Glacial ice	Ny-Ålesund, Svalbard, Norway
EXF-3378	*Aureobasidium melanogenum*	Water fountain	Bangkok, Thailand
EXF-15	*Hortaea werneckii*	Brine from solar saltern	Santa pola, Spain
EXF-562	*Hortaea werneckii*	Soil on the seacoast	Namibia
EXF-2000	*Hortaea werneckii*	Hypersaline water, solar saltern	Sečovlje, Slovenia

**Table 2:** Comparison of linear models used to describe the effects of salinity and temperature on the doubling time of *Aureobasidium* spp., evaluated using Akaike’s Information Criterion (AIC).

*Aureobasidium* spp.				
Linear Model	df	AIC	ΔAIC	Weight
log(Doubling Time) ~ Species × NaCl × Temperature	13	375.32	0	1
log(Doubling Time) ~ NaCl × Temperature	5	508.93	133.61	9.69 × 10^−30^
log(Doubling Time) ~ NaCl + Temperature	4	517.32	142	1.46 × 10^−31^
log(Doubling Time) ~ NaCl	3	533.25	157.93	5.08 × 10^−35^
log(Doubling Time) ~ Temperature	3	848.82	473.51	1.51 × 10^−103^
log(Doubling Time) ~ Null (intercept)	2	860.66	485.35	4.06 × 10^−106^

**Table 3:** Comparison of linear models used to describe the effects of salinity and temperature on the doubling time of *Hortaea werneckii* (all strains), evaluated using Akaike’s Information Criterion (AIC).

*Hortaea werneckii* (All Strains)				
Linear Model	df	AIC	ΔAIC	Weight
log(Doubling Time) ~ Ploidy × NaCl	5	514.36	0	8.09 × 10^−1^
log(Doubling Time) ~ Ploidy × (NaCl + Temperature)	7	517.65	3.28	1.57 × 10^−1^
log(Doubling Time) ~ Ploidy × NaCl × Temperature	9	520.17	6.35	3.38 × 10^−2^
log(Doubling Time) ~ NaCl	3	626.86	112.5	3.02 × 10^−25^
log(Doubling Time) ~ NaCl + Temperature	4	628.85	114.49	1.11 × 10^−25^
log(Doubling Time) ~ NaCl × Temperature	5	630.8	116.44	4.21 × 10^−26^
log(Doubling Time) ~ Null (intercept)	2	687.47	173.11	2.08 × 10^−38^
log(Doubling Time) ~ Temperature	3	689.38	175.02	8.01 × 10^−39^

## Data Availability

The original contributions presented in this study are included in the article/Supplementary Material. Further inquiries can be directed to the corresponding author.

## Supplementary Materials

The following supporting information can be downloaded at: https://www.mdpi.com/article/10.3390/jof12010043/s1, Table S1: Survival of short-term (1 day) freezing and desiccation in *Aureobasidium pullulans* (EXF-150) following four preconditioning regimes; Table S2: Survival of long-term (1 week) freezing and desiccation in *Aureobasidium pullulans* (EXF-150) following four preconditioning regimes; Table S3: Survival of short-term (1 day) freezing and desiccation in *Hortaea werneckii* (EXF-2000) following four preconditioning regimes; Table S4: Survival of long-term (1 week) freezing and desiccation in *Hortaea werneckii* (EXF-2000) following four preconditioning regimes; Table S5: Mean doubling times and standard deviations of *Aureobasidium pullulans* (EXF-150) at different combinations of temperature and NaCl concentration; Table S6: Mean doubling times and standard deviations of *Aureobasidium pullulans* (EXF-3645) at different combinations of temperature and NaCl concentration; Table S7: Mean doubling times and standard deviations of *Aureobasidium subglaciale* (EXF-2481) at different combinations of temperature and NaCl concentration; Table S8: Mean doubling times and corresponding standard deviations of *Aureobasidium melanogenum* (EXF-3378) at different temperatures and NaCl concentrations; Table S9: Mean doubling times and corresponding standard deviations of *Hortaea werneckii* (EXF-15) at different temperatures and NaCl concentrations; Table S10: Mean doubling times and corresponding standard deviations of *Hortaea werneckii* (EXF-562) at different temperatures and NaCl concentrations; Table S11: Mean doubling times and corresponding standard deviations of *Hortaea werneckii* (EXF-2000) at different temperatures and NaCl concentrations; Table S12: AIC-based model comparisons for linear models of log(Doubling Time) across temperature and NaCl concentration for each *Aureobasidium* and *Hortaea werneckii* strain.

## Abbreviations

The following abbreviations are used in this manuscript:

EXF	Designation of fungal strains from the Ex Culture Collection of the Infrastructural Centre Mycosmo, MRIC UL, Slovenia (Department of Biology, Biotechnical Faculty, University of Ljubljana)
a_w_	water activity
ROS	Reactive oxygen species
CFU	Colony-forming units
OD_600_	Optical density measured at 600 nm
AIC	Akaike’s Information Criterion
YNB-Glc	Yeast nitrogen base supplemented with glucose
