# Supplementary material for: Stress-Induced Cross-Protection and Combined Stress Responses in Extremotolerant Black Yeasts
Source: J Fungi (Basel). 2026 Jan 6;12(1):43. doi: 10.3390/jof12010043 (PMC12842981; doi:10.3390/jof12010043)
Supplement: Supplementary file 1 [file jof-12-00043-s001.zip › jof-4016892-supplementary.pdf]

Table S1: Survival of short-term (1 day) freezing and desiccation in *Aureobasidium pullulans* (EXF-150) following four preconditioning regimes.

	Preconditioning	Initial cell count (CFU/mL)	Cell count after freezing (CFU/mL)	Cell count after desiccation (CFU/mL)	Freezing survival (%)	Freezing death rate (%)	Desiccation survival (%)	Desiccation death rate (%)
***Aureobasidium pullulans* (EXF-150)**	Control (25 °C)	2.90E+06	2.83E+06	2.92E+06	97.7	2.3	100.8	-0.8
		3.40E+06	3.33E+06	3.42E+06	97.9	2.1	100.5	-0.5
		3.90E+06	3.68E+06	3.92E+06	94.3	5.7	100.5	-0.5
		4.58E+06	4.32E+06	4.60E+06	94.3	5.7	100.5	-0.5
		3.30E+06	3.33E+06	2.79E+06	100.8	-0.8	84.5	15.5
		3.88E+06	3.91E+06	3.27E+06	100.8	-0.8	84.3	15.7
	Cold (15 °C)	2.89E+06	2.74E+06	3.00E+06	94.8	5.2	103.8	-3.8
		3.39E+06	3.22E+06	3.52E+06	95.0	5.0	103.8	-3.8
		1.90E+06	1.90E+06	1.82E+06	99.8	0.2	95.6	4.4
		2.24E+06	2.24E+06	2.14E+06	100.2	-0.2	95.7	4.3
		3.39E+06	3.39E+06	3.37E+06	100.1	-0.1	99.5	0.5
		3.97E+06	4.00E+06	3.95E+06	100.6	-0.6	99.4	0.6
	17% NaCl (w/v)	2.92E+06	2.90E+06	3.01E+06	99.4	0.6	103.2	-3.2
		3.42E+06	3.40E+06	3.53E+06	99.3	0.7	103.1	-3.1
		2.71E+06	2.73E+06	2.85E+06	100.6	-0.6	105.0	-5.0
		3.19E+06	3.21E+06	3.35E+06	100.8	-0.8	105.1	-5.1
		3.66E+06	3.56E+06	3.24E+06	97.2	2.8	88.5	11.5
		4.30E+06	4.18E+06	3.80E+06	97.2	2.8	88.4	11.6
	17% NaCl (w/v) + Cold (15 °C)	3.28E+06	3.37E+06	3.83E+06	102.9	-2.9	116.9	-16.9
		3.84E+06	3.95E+06	4.49E+06	102.7	-2.7	116.8	-16.8
		4.84E+06	4.79E+06	4.88E+06	99.0	1.0	100.8	-0.8
		5.68E+06	5.63E+06	5.72E+06	99.1	0.9	100.7	-0.7
		3.53E+06	3.60E+06	3.77E+06	101.9	-1.9	106.7	-6.7
		4.15E+06	4.23E+06	4.43E+06	102.0	-2.0	106.8	-6.8

Table S2: Survival of long-term (1 week) freezing and desiccation in *Aureobasidium pullulans* (EXF-150) following four preconditioning regimes.

	Preconditioning	Initial cell count (CFU/mL)	Cell count after freezing (CFU/mL)	Cell count after desiccation (CFU/mL)	Freezing survival (%)	Freezing death rate (%)	Desiccation survival (%)	Desiccation death rate (%)
***Aureobasidium pullulans* (EXF-150)**	Control (25 °C)	2.01E+06	1.02E+06	1.90E+05	50.7	49.3	9.5	90.5
		2.27E+06	1.14E+06	2.24E+05	50.2	49.8	9.9	90.1
		1.06E+06	1.42E+06	2.10E+05	134.0	-34.0	19.8	80.2
		1.51E+06	1.58E+06	2.42E+05	104.6	-4.6	16.0	84.0
		1.75E+06	1.66E+06	1.90E+05	94.9	5.1	10.9	89.1
		1.82E+06	1.84E+06	2.16E+05	101.1	-1.1	11.9	88.1
	Cold (15 °C)	1.85E+06	1.82E+06	1.56E+06	98.4	1.6	84.3	15.7
		2.15E+06	2.14E+06	1.84E+06	99.5	0.5	85.6	14.4
		1.80E+06	1.45E+06	1.82E+06	80.6	19.4	101.1	-1.1
		2.14E+06	1.69E+06	2.14E+06	79.0	21.0	100.0	0.0
		1.55E+06	1.55E+06	1.53E+06	100.0	0.0	98.7	1.3
		1.81E+06	1.81E+06	1.79E+06	100.0	0.0	98.9	1.1
	17% NaCl (w/v)	1.86E+06	1.96E+06	2.09E+06	105.4	-5.4	112.4	-12.4
		2.28E+06	2.30E+06	2.45E+06	100.9	-0.9	107.5	-7.5
		2.37E+06	2.46E+06	2.85E+06	103.8	-3.8	120.3	-20.3
		2.89E+06	2.88E+06	3.35E+06	99.7	0.3	115.9	-15.9
		1.78E+06	2.64E+06	2.58E+06	148.3	-48.3	144.9	-44.9
		2.18E+06	3.10E+06	3.02E+06	142.2	-42.2	138.5	-38.5
	17% NaCl (w/v) + Cold (15 °C)	3.88E+06	4.32E+06	3.96E+06	111.4	-11.4	102.1	-2.1
		4.73E+06	5.28E+06	4.64E+06	111.6	-11.6	98.1	1.9
		4.59E+06	4.60E+06	3.04E+06	100.2	-0.2	66.2	33.8
		5.61E+06	5.00E+06	3.56E+06	89.1	10.9	63.5	36.5
		2.70E+06	2.80E+06	4.14E+06	103.7	-3.7	153.3	-53.3
		3.30E+06	3.42E+06	4.86E+06	103.7	-3.7	147.3	-47.3

Table S3: Survival of short-term (1 day) freezing and desiccation in *Hortaea werneckii* (EXF-2000) following four preconditioning regimes.

	Preconditioning	Initial cell count (CFU/mL)	Cell count after freezing (CFU/mL)	Cell count after desiccation (CFU/mL)	Freezing survival (%)	Freezing death rate (%)	Desiccation survival (%)	Desiccation death rate (%)
***Hortaea werneckii* (EXF-2000)**	Control (25 °C)	3.10E+06	2.91E+06	2.86E+06	93.9	6.1	92.3	7.7
		3.40E+06	3.41E+06	3.36E+06	100.3	-0.3	98.8	1.2
		3.04E+06	3.10E+06	3.36E+06	102.0	-2.0	110.5	-10.5
		3.58E+06	3.64E+06	3.94E+06	101.7	-1.7	110.1	-10.1
		3.16E+06	3.00E+06	3.16E+06	94.9	5.1	100.0	0.0
		3.70E+06	3.52E+06	3.70E+06	95.1	4.9	100.0	0.0
	Cold (15 °C)	3.91E+06	3.61E+06	3.77E+06	92.3	7.7	96.4	3.6
		4.59E+06	4.23E+06	4.43E+06	92.2	7.8	96.5	3.5
		3.89E+06	3.78E+06	4.15E+06	97.2	2.8	106.7	-6.7
		4.57E+06	4.44E+06	4.87E+06	97.2	2.8	106.6	-6.6
		4.20E+06	3.99E+06	4.20E+06	95.0	5.0	100.0	0.0
		4.92E+06	4.69E+06	4.93E+06	95.3	4.7	100.2	-0.2
	NaCl (25%)	2.99E+06	2.89E+06	3.24E+06	96.7	3.3	108.4	-8.4
		3.53E+06	3.39E+06	3.80E+06	96.0	4.0	107.6	-7.6
		3.24E+06	3.26E+06	2.94E+06	100.6	-0.6	90.7	9.3
		3.80E+06	3.82E+06	3.45E+06	100.5	-0.5	90.8	9.2
		3.20E+06	3.16E+06	3.18E+06	98.8	1.3	99.4	0.6
		3.76E+06	3.70E+06	3.74E+06	98.4	1.6	99.5	0.5
	25% NaCl (w/v) + Cold (15 °C)	3.26E+06	3.66E+06	2.98E+06	112.3	-12.3	91.4	8.6
		3.82E+06	4.30E+06	3.50E+06	112.6	-12.6	91.6	8.4
		3.29E+06	3.97E+06	3.55E+06	120.7	-20.7	107.9	-7.9
		3.79E+06	4.67E+06	4.17E+06	123.2	-23.2	110.0	-10.0
		3.64E+06	3.51E+06	3.60E+06	96.4	3.6	98.9	1.1
		4.28E+06	4.11E+06	4.22E+06	96.0	4.0	98.6	1.4

Table S4: Survival of long-term (1 week) freezing and desiccation in *Hortaea werneckii* (EXF-2000) following four preconditioning regimes.

	Preconditioning	Initial cell count (CFU/mL)	Cell count after freezing (CFU/mL)	Cell count after desiccation (CFU/mL)	Freezing survival (%)	Freezing death rate (%)	Desiccation survival (%)	Desiccation death rate (%)
***Hortaea werneckii* (EXF-2000)**	Control (25 °C)	1.15E+06	1.99E+06	1.79E+05	173.0	-73.0	15.6	84.4
		1.35E+06	2.33E+06	2.11E+05	172.6	-72.6	15.6	84.4
		1.21E+06	1.81E+06	2.44E+05	149.6	-49.6	20.2	79.8
		1.42E+06	2.13E+06	2.86E+05	150.0	-50.0	20.1	79.9
		1.32E+06	1.49E+06	2.25E+05	112.9	-12.9	17.0	83.0
		1.54E+06	1.75E+06	2.65E+05	113.6	-13.6	17.2	82.8
	Cold (15 °C)	2.95E+06	3.61E+06	3.77E+06	122.4	-22.4	127.8	-27.8
		3.47E+06	4.23E+06	4.43E+06	121.9	-21.9	127.7	-27.7
		1.77E+06	1.94E+05	2.31E+06	11.0	89.0	130.5	-30.5
		2.08E+06	2.28E+05	2.71E+06	11.0	89.0	130.3	-30.3
		1.57E+06	1.23E+06	1.44E+06	78.3	21.7	91.7	8.3
		1.85E+06	1.45E+06	1.68E+06	78.4	21.6	90.8	9.2
	NaCl (25%)	1.09E+06	1.97E+06	1.56E+06	180.7	-80.7	143.1	-43.1
		1.27E+06	2.31E+06	1.84E+06	181.9	-81.9	144.9	-44.9
		1.35E+06	9.57E+05	2.93E+06	70.9	29.1	217.0	-117.0
		1.59E+06	1.12E+06	3.45E+06	70.4	29.6	217.0	-117.0
		1.51E+06	1.32E+06	8.83E+05	87.4	12.6	58.5	41.5
		1.77E+06	1.54E+06	1.04E+06	87.0	13.0	58.8	41.2
	25% NaCl (w/v) + Cold (15 °C)	2.34E+06	1.82E+06	4.64E+06	77.8	22.2	198.3	-98.3
		2.74E+06	2.14E+06	5.44E+06	78.1	21.9	198.5	-98.5
		3.36E+06	3.97E+06	3.55E+06	118.2	-18.2	105.7	-5.7
		3.95E+06	4.67E+06	4.17E+06	118.2	-18.2	105.6	-5.6
		1.59E+06	1.33E+06	1.85E+06	83.6	16.4	116.4	-16.4
		1.87E+06	1.57E+06	2.17E+06	84.0	16.0	116.0	-16.0

Table S5: Mean doubling times and standard deviations of *Aureobasidium pullulans* (EXF-150) at different combinations of temperature and NaCl concentration.

Aureobasidium pullulans (EXF-150)			
**Temperature (°C)**	**NaCl (%)**	**Doubling time (h, mean)**	**Doubling time (h, SD)**
15	0	10.0	0.2
15	2	10.2	0.2
15	4	13.6	0.4
15	6	18.4	1.0
15	8	24.1	1.3
15	10	31.3	1.3
15	12	41.8	1.4
15	14	67.1	3.9
15	16	NA	NA
15	17	NA	NA
20	0	7.7	0.2
20	2	9.7	0.1
20	4	12.0	0.5
20	6	14.1	0.6
20	8	16.4	1.6
20	10	20.4	1.8
20	12	29.6	2.7
20	14	39.6	3.7
20	16	NA	NA
20	17	NA	NA
25	0	6.8	0.1
25	2	8.6	0.2
25	4	10.2	0.3
25	6	11.6	0.8
25	8	14.2	0.8
25	10	17.9	1.2
25	12	27.0	1.7
25	14	41.7	8.4
25	16	NA	NA
25	17	NA	NA
30	0	7.8	0.2
30	2	8.0	0.2
30	4	9.3	0.3
30	6	10.9	0.4
30	8	14.1	0.8
30	10	18.1	1.2
30	12	25.0	1.6
30	14	32.9	8.1
30	16	NA	NA
30	17	NA	NA
35	0	NA	NA
35	2	NA	NA

35	4	NA	NA
35	6	NA	NA
35	8	NA	NA
35	10	NA	NA
35	12	NA	NA
35	14	NA	NA
35	16	NA	NA
35	17	NA	NA
37	0	NA	NA
37	2	NA	NA
37	4	NA	NA
37	6	NA	NA
37	8	NA	NA
37	10	NA	NA
37	12	NA	NA
37	14	NA	NA
37	16	NA	NA
37	17	NA	NA

Table S6: Mean doubling times and standard deviations of *Aureobasidium pullulans* (EXF-3645) at different combinations of temperature and NaCl concentration.

Aureobasidium pullulans (EXF-3645)			
**Temperature (°C)**	**NaCl (%)**	**Doubling time (h, mean)**	**Doubling time (h, SD)**
15	0	7.1	0.9
15	2	10.3	1.3
15	4	11.0	1.5
15	6	12.4	2.0
15	8	15.1	3.5
15	10	20.2	2.7
15	12	27.9	4.1
15	14	45.9	5.8
15	16	NA	NA
15	17	NA	NA
20	0	7.4	1.2
20	2	7.2	1.1
20	4	8.5	1.2
20	6	9.4	1.4
20	8	11.6	0.1
20	10	13.4	1.1
20	12	16.9	1.5
20	14	25.0	4.5
20	16	32.5	0.4
20	17	NA	NA
25	0	6.2	0.1
25	2	7.2	0.1
25	4	8.2	0.2

25	6	9.4	1.0
25	8	11.5	0.8
25	10	17.6	0.7
25	12	24.8	1.4
25	14	30.5	3.7
25	16	53.3	6.4
25	17	NA	NA
30	0	7.7	0.1
30	2	7.7	0.2
30	4	9.1	0.2
30	6	11.1	0.3
30	8	13.8	1.2
30	10	18.2	0.8
30	12	23.7	1.3
30	14	25.4	1.0
30	16	NA	NA
30	17	NA	NA
35	0	NA	NA
35	2	NA	NA
35	4	NA	NA
35	6	NA	NA
35	8	NA	NA
35	10	NA	NA
35	12	NA	NA
35	14	NA	NA
35	16	NA	NA
35	17	NA	NA
37	0	NA	NA
37	2	NA	NA
37	4	NA	NA
37	6	NA	NA
37	8	NA	NA
37	10	NA	NA
37	12	NA	NA
37	14	NA	NA
37	16	NA	NA
37	17	NA	NA

Table S7: Mean doubling times and standard deviations of *Aureobasidium subglaciale* (EXF-2481) at different combinations of temperature and NaCl concentration.

Aureobasidium subglaciale (EXF-2481)			
**Temperature (°C)**	**NaCl (%)**	**Doubling time (h, mean)**	**Doubling time (h, SD)**
15	0	11.8	0.1
15	2	13.0	0.4
15	4	14.9	0.1
15	6	19.3	0.1

15	8	24.9	0.7
15	10	36.0	2.2
15	12	52.2	1.1
15	14	NA	NA
15	16	NA	NA
15	17	NA	NA
20	0	8.5	0.3
20	2	10.0	0.1
20	4	11.7	0.8
20	6	12.4	1.0
20	8	14.0	0.4
20	10	17.2	0.8
20	12	26.5	1.6
20	14	36.2	3.2
20	16	36.6	0.4
20	17	105.2	5.3
25	0	8.5	0.0
25	2	9.9	0.2
25	4	11.8	0.0
25	6	13.9	0.2
25	8	14.2	0.7
25	10	18.3	0.3
25	12	25.2	0.8
25	14	NA	NA
25	16	NA	NA
25	17	NA	NA
30	0	30.1	0.9
30	2	30.9	1.3
30	4	42.6	5.1
30	6	304.0	27.5
30	8	NA	NA
30	10	NA	NA
30	12	NA	NA
30	14	NA	NA
30	16	NA	NA
30	17	NA	NA
35	0	NA	NA
35	2	NA	NA
35	4	NA	NA
35	6	NA	NA
35	8	NA	NA
35	10	NA	NA
35	12	NA	NA
35	14	NA	NA
35	16	NA	NA
35	17	NA	NA

37	0	NA	NA
37	2	NA	NA
37	4	NA	NA
37	6	NA	NA
37	8	NA	NA
37	10	NA	NA
37	12	NA	NA
37	14	NA	NA
37	16	NA	NA
37	17	NA	NA

Table S8: Mean doubling times and corresponding standard deviations of *Aureobasidium melanogenum* (EXF-3378) at different temperatures and NaCl concentrations.

Aureobasidium melanogenum (EXF-3378)			
**Temperature (°C)**	**NaCl (%)**	**Doubling time (h, mean)**	**Doubling time (h, SD)**
15	0	12.9	0.0
15	2	19.0	0.2
15	4	24.4	1.2
15	6	26.5	1.1
15	8	30.6	1.3
15	10	39.6	3.3
15	12	66.4	9.6
15	14	106.7	2.5
15	16	NA	NA
15	17	NA	NA
20	0	5.9	0.1
20	2	8.4	0.1
20	4	8.5	0.4
20	6	9.8	0.2
20	8	12.4	0.1
20	10	15.4	0.7
20	12	20.9	0.5
20	14	34.6	1.4
20	16	69.0	35.6
20	17	NA	NA
25	0	6.4	0.1
25	2	7.4	0.0
25	4	8.4	0.1
25	6	9.5	0.1
25	8	10.3	0.2
25	10	12.6	0.1
25	12	17.8	0.4
25	14	48.8	4.6
25	16	109.2	3.4
25	17	NA	NA
30	0	6.3	0.3

30	2	7.5	0.1
30	4	8.4	0.0
30	6	12.8	2.1
30	8	14.5	0.9
30	10	20.4	1.7
30	12	25.1	6.2
30	14	43.2	11.8
30	16	NA	NA
30	17	NA	NA
35	0	7.0	0.2
35	2	8.0	1.3
35	4	10.0	0.4
35	6	13.7	0.8
35	8	13.9	0.2
35	10	16.1	0.5
35	12	18.6	0.1
35	14	NA	NA
35	16	NA	NA
35	17	NA	NA
37	0	NA	NA
37	2	NA	NA
37	4	NA	NA
37	6	NA	NA
37	8	NA	NA
37	10	NA	NA
37	12	NA	NA
37	14	NA	NA
37	16	NA	NA
37	17	NA	NA

Table S9: Mean doubling times and corresponding standard deviations of *Hortaea werneckii* (EXF-15) at different temperatures and NaCl concentrations.

Hortaea werneckii (EXF-15)			
**Temperature (°C)**	**NaCl (%)**	**Doubling time (h, mean)**	**Doubling time (h, SD)**
15	0	23.8	1.2
15	5	25.1	0.3
15	10	31.1	0.6
15	12.5	38.4	0.3
15	15	64.1	8.2
15	17.5	NA	NA
15	20	NA	NA
15	22.5	NA	NA
15	25	NA	NA
15	27.5	NA	NA
20	0	12.6	0.1
20	5	16.0	1.1

20	10	19.6	1.6
20	12.5	22.9	2.1
20	15	26.8	2.1
20	17.5	32.2	2.7
20	20	42.5	1.4
20	22.5	69.1	3.2
20	25	95.2	11.6
20	27.5	NA	NA
25	0	12.6	0.3
25	5	14.4	0.4
25	10	19.5	2.2
25	12.5	24.9	5.8
25	15	28.9	6.2
25	17.5	32.8	4.9
25	20	47.1	9.8
25	22.5	59.0	2.7
25	25	88.9	32.6
25	27.5	NA	NA
30	0	9.5	0.4
30	5	11.8	0.5
30	10	15.5	1.6
30	12.5	23.0	1.8
30	15	23.4	3.6
30	17.5	27.6	1.0
30	20	42.7	8.4
30	22.5	53.6	3.4
30	25	NA	NA
30	27.5	NA	NA
35	0	15.6	0.2
35	5	18.0	0.2
35	10	29.3	0.6
35	12.5	39.0	1.8
35	15	61.0	2.8
35	17.5	120.3	14.5
35	20	187.9	20.4
35	22.5	NA	NA
35	25	NA	NA
35	27.5	NA	NA
37	0	NA	NA
37	5	NA	NA
37	10	NA	NA
37	12.5	NA	NA
37	15	NA	NA
37	17.5	NA	NA
37	20	NA	NA
37	22.5	NA	NA

37	25	NA	NA
37	27.5	NA	NA

Table S10: Mean doubling times and corresponding standard deviations of *Hortaea werneckii* (EXF-562) at different temperatures and NaCl concentrations.

Hortaea werneckii (EXF-562)			
**Temperature (°C)**	**NaCl (%)**	**Doubling time (h, mean)**	**Doubling time (h, SD)**
15	0	24.1	0.2
15	5	23.6	0.2
15	10	30.1	0.2
15	12.5	38.4	0.6
15	15	45.9	0.6
15	17.5	48.7	6.5
15	20	NA	NA
15	22.5	NA	NA
15	25	NA	NA
15	27.5	NA	NA
20	0	13.1	0.2
20	5	16.5	0.2
20	10	20.7	0.5
20	12.5	25.5	0.6
20	15	30.0	1.0
20	17.5	37.3	1.3
20	20	51.3	1.9
20	22.5	59.7	2.5
20	25	81.2	4.8
20	27.5	NA	NA
25	0	10.0	0.2
25	5	13.6	0.5
25	10	27.1	0.3
25	12.5	26.8	1.3
25	15	27.0	0.5
25	17.5	35.2	3.4
25	20	48.6	5.2
25	22.5	61.8	8.7
25	25	80.0	14.8
25	27.5	NA	NA
30	0	10.7	0.6
30	5	14.7	0.4
30	10	19.0	0.9
30	12.5	26.4	2.1
30	15	27.7	0.8
30	17.5	36.0	0.2
30	20	45.2	2.0
30	22.5	60.6	12.1
30	25	NA	NA

30	27.5	NA	NA
35	0	15.8	0.1
35	5	15.4	1.1
35	10	20.8	0.4
35	12.5	31.6	0.7
35	15	45.0	4.1
35	17.5	NA	NA
35	20	NA	NA
35	22.5	NA	NA
35	25	NA	NA
35	27.5	NA	NA
37	0	40.8	1.4
37	5	NA	NA
37	10	NA	NA
37	12.5	NA	NA
37	15	NA	NA
37	17.5	NA	NA
37	20	NA	NA
37	22.5	NA	NA
37	25	NA	NA
37	27.5	NA	NA

Table S11: Mean doubling times and corresponding standard deviations of *Hortaea werneckii* (EXF-2000) at different temperatures and NaCl concentrations.

Hortaea werneckii (EXF-2000)			
**Temperature (°C)**	**NaCl (%)**	**Doubling time (h, mean)**	**Doubling time (h, SD)**
15	0	21.5	0.2
15	5	29.4	0.1
15	10	40.7	0.2
15	12.5	51.7	1.2
15	15	50.3	2.0
15	17.5	78.5	12.0
15	20	115.8	7.3
15	22.5	NA	NA
15	25	NA	NA
15	27.5	NA	NA
20	0	14.5	0.1
20	5	16.8	0.1
20	10	20.3	0.2
20	12.5	24.6	0.5
20	15	28.6	0.8
20	17.5	35.5	0.1
20	20	46.4	0.6
20	22.5	58.2	2.0
20	25	88.8	19.9
20	27.5	NA	NA

25	0	10.7	0.2
25	5	14.4	0.3
25	10	20.3	0.7
25	12.5	24.7	2.8
25	15	25.6	1.5
25	17.5	29.5	2.4
25	20	42.2	3.8
25	22.5	48.7	6.0
25	25	59.7	2.7
25	27.5	NA	NA
30	0	10.1	0.1
30	5	12.7	0.1
30	10	18.5	0.3
30	12.5	23.0	0.4
30	15	27.0	1.2
30	17.5	32.8	2.2
30	20	51.1	1.9
30	22.5	112.8	15.6
30	25	NA	NA
30	27.5	NA	NA
35	0	17.1	0.1
35	5	21.4	0.4
35	10	29.8	1.0
35	12.5	40.1	1.0
35	15	59.9	1.2
35	17.5	105.7	9.1
35	20	NA	NA
35	22.5	NA	NA
35	25	NA	NA
35	27.5	NA	NA
37	0	104.3	9.6
37	5	210.1	7.5
37	10	NA	NA
37	12.5	NA	NA
37	15	NA	NA
37	17.5	NA	NA
37	20	NA	NA
37	22.5	NA	NA
37	25	NA	NA
37	27.5	NA	NA

Table S12: AIC-based model comparisons for linear models of log(Doubling Time) across temperature and NaCl concentration for each *Aureobasidium* and *Hortaea werneckii* strain.

Aureobasidium pullulans (EXF-150)				
**Linear model**	**df**	**AIC**	**ΔAIC**	**Weight**
log(Doubling Time) ~ NaCl × Temperature	5	-99.12	0	9.74E-01
log(Doubling Time) ~ NaCl + Temperature	4	-91.85	7.27	2.58E-02
log(Doubling Time) ~ NaCl	3	-10.39	88.73	5.26E-20
log(Doubling Time) ~ Temperature	3	168.57	267.68	7.27E-59
log(Doubling Time) ~ Null (intercept)	2	174.84	273.96	3.15E-60
Aureobasidium pullulans (EXF-3645)				
**Linear model**	**df**	**AIC**	**ΔAIC**	**Weight**
log(Doubling Time) ~ NaCl + Temperature	4	-29.33	0	5.87E-01
log(Doubling Time) ~ NaCl × Temperature	5	-27.36	1.97	2.20E-01
log(Doubling Time) ~ NaCl	3	-27.1	2.23	1.93E-01
log(Doubling Time) ~ Null (intercept)	2	180.86	210.18	1.34E-46
log(Doubling Time) ~ Temperature	3	182.33	211.65	6.44E-47
Aureobasidium subglaciale (EXF-2481)				
**Linear model**	**df**	**AIC**	**ΔAIC**	**Weight**
log(Doubling Time) ~ NaCl × Temperature	5	163.62	0	8.13E-01
log(Doubling Time) ~ NaCl + Temperature	4	166.56	2.94	1.87E-01
log(Doubling Time) ~ NaCl	3	178.71	15.09	4.29E-04
log(Doubling Time) ~ Temperature	3	199.65	36.03	1.22E-08
log(Doubling Time) ~ Null (intercept)	2	201.94	38.32	3.89E-09
Aureobasidium melanogenum (EXF-3378)				
**Linear model**	**df**	**AIC**	**ΔAIC**	**Weight**
log(Doubling Time) ~ NaCl × Temperature	5	110.4	0	5.52E-01
log(Doubling Time) ~ NaCl + Temperature	4	110.82	0.42	4.48E-01
log(Doubling Time) ~ NaCl	3	150.91	40.51	8.01E-10
log(Doubling Time) ~ Temperature	3	263.55	153.15	3.07E-34
log(Doubling Time) ~ Null (intercept)	2	280.78	170.38	5.55E-38
Hortaea werneckii (EXF-15); haploid				
**Linear model**	**df**	**AIC**	**ΔAIC**	**Weight**
log(Doubling Time) ~ NaCl × Temperature	5	104.16	0.00	9.73E-01
log(Doubling Time) ~ NaCl	3	111.96	7.80	1.97E-02
log(Doubling Time) ~ NaCl + Temperature	4	113.89	9.73	7.50E-03
log(Doubling Time) ~ Null (intercept)	2	229.77	125.61	5.14E-28
log(Doubling Time) ~ Temperature	3	231.70	127.55	1.96E-28
Hortaea werneckii (EXF-562); haploid				
**Linear model**	**df**	**AIC**	**ΔAIC**	**Weight**
log(Doubling Time) ~ NaCl × Temperature	5	32.72	0.00	6.30E-01
log(Doubling Time) ~ NaCl	3	34.81	2.09	2.22E-01
log(Doubling Time) ~ NaCl + Temperature	4	35.61	2.88	1.49E-01
log(Doubling Time) ~ Temperature	3	184.01	151.28	8.87E-34
log(Doubling Time) ~ Null (intercept)	2	185.49	152.76	4.23E-34
Hortaea werneckii (EXF-2000); diploid				
**Linear model**	**df**	**AIC**	**ΔAIC**	**Weight**
log(Doubling Time) ~ NaCl × Temperature	5	213.06	0	9.71E-01

log(Doubling Time) ~ NaCl + Temperature	4	220.67	7.61	2.16E-02
log(Doubling Time) ~ NaCl	3	222.9	9.84	7.08E-03
log(Doubling Time) ~ Null (intercept)	2	266.76	53.7	2.12E-12
log(Doubling Time) ~ Temperature	3	268.42	55.36	9.25E-13
